# Fostering disability-inclusive HIV/AIDS programs in northeast India: a participatory study

**DOI:** 10.1186/1471-2458-7-125

**Published:** 2007-06-26

**Authors:** Martha Morrow, MC Arunkumar, Emma Pearce, Heather E Dawson

**Affiliations:** 1 Nossal Institute of Global Health, University of Melbourne, Melbourne, Vic 3010, Australia; 2 Department of Anthropology, Manipur University, Imphal, Manipur, India; 3 School of Population Health, University of Melbourne, Melbourne, Vic 3010, Australia

## Abstract

**Background:**

Manipur and Nagaland in northeast India are among the Indian states with the highest prevalence of HIV. Most prevention and care programs focus on identified "high risk" groups, but recent data suggest the epidemic is increasing among the general population, primarily through heterosexual sex. People with disability (PWD) in India are more likely than the general population to be illiterate, unemployed and impoverished, but little is known of their HIV risk.

**Methods:**

This project aimed to enable HIV programs in Manipur and Nagaland to be more disability-inclusive. The objectives were to: explore HIV risk and risk perception in relation to PWD among HIV and disability programmers, and PWD themselves; identify HIV-related education and service needs and preferences of PWD; and utilise findings and stakeholder consultation to draft practical guidelines for inclusion of disability into HIV programming. Data were collected through a survey and several qualitative tools.

**Results:**

The findings revealed that participants believe PWD in these states are potentially vulnerable to HIV transmission due to social exclusion and poverty, lack of knowledge, gender norms and obstacles to accessing HIV programs. Neither HIV nor disability organisations currently address the risks, needs and preferences of PWD.

**Conclusion:**

The Guidelines produced in the project and disseminated to stakeholders emphasise opportunities for taking action with minimal cost and resources, such as using the networks and expertise of both HIV and disability sectors, producing HIV material in a variety of formats, and promoting accessibility to mainstream HIV education and services. The human rights obligations and public health benefits of modifying national and state policies and programs to assist this highly disadvantaged population are also highlighted.

## Background

Evidence suggests that in developing countries poverty and a lack of economic and educational opportunities, as often experienced by people with disability (PWD), influence HIV vulnerability [1-4]. Despite this theoretical link, there is a dearth of research that documents HIV prevalence, risk and vulnerability among PWD, the adequacy of service provision, and the extent to which HIV programs have addressed their needs. The 2001 census identified that 2.2% of the Indian population were living with a disability [5]. However, organisations working in the field of disability claim that the strong stigmatisation of disability in Indian society results in an underestimation of its prevalence. Disability activists, NGOs and many government agencies estimate the real prevalence to be 5–6% of the total population [6,7].

While an increasing body of research into the HIV risk and vulnerability among PWD has originated from the African continent [8-13], there has been a gap in research from India. However, the theoretical assumption about potential HIV vulnerability among PWD clearly applies in India, where poverty is considered the principal cause of disability, and where the disabled too often lack opportunities for education and social advancement [7].

Debate continues about whether or not India has overtaken South Africa in having the largest number of people living with HIV in any country [14]. However, the fact remains that the progression of India's epidemic has great relevance for international control because of the sheer size of its population (1.1 billion). The official estimated prevalence is 0.91% among people aged 15–49 [15], and in 2005 there were approximately 5.7 million people living with HIV in India [16]. However, prevalence rates vary greatly across the vast sub-continent.

Manipur and Nagaland, northeastern states that abut Burma, are categorised as 'high prevalence' (>1% HIV positivity among women in prenatal clinics and >5% among patients at government sexually-transmitted diseases clinics). A recent review by Chandrasekaran et al. [17] cites prevalence estimates in these states among 'high-risk' groups of 0.4 – 33.6% for injecting drug users (IDUs) and 4 – 29.7% for female sex workers. Whilst injection drug use – practised by 1.9 – 2.7% of the adult population in these states – is the dominant mode of transmission, further spread into the wider community is occurring through regular partners and clients of sex workers (often married men, as well as interstate truckers) and sexual partners of IDUs [17]. These states are populated overwhelmingly by a number of distinct ethnic minority (or tribal) groups which speak a variety of languages. They are characterised by high unemployment and weak infrastructure, both of which are linked to longstanding and multiple insurgencies. There are reports of underground groups extorting funds intended for HIV prevention and care in Manipur [18] and even conducting executions of IDUs [19].

The National AIDS Control Programme (NACP) provides the national strategy for India's HIV response, with implementation of projects at state level under the coordination of State AIDS Control Societies [17]. There has also been a significant response from non-governmental organisations (NGOs). Projects extend from mainstream awareness and education campaigns, care and support for people living with HIV/AIDS, to working with marginalised populations [20,21].

Despite the catch-cry that 'everyone can be at risk of HIV' there is little or no recognition that PWD may be at risk and, if so, have special needs. While generic guidelines with this focus have recently been published [22] following an international survey, there is nothing similar designed specifically for India. India's National AIDS Control Organisation has not explicitly cited PWD as a vulnerable group, and their national programs and policies have not targeted them. The launch of the third program, NACP III (2007 – 2011), offers a superb opportunity to incorporate the needs of PWD into existing and new approaches. There are similar opportunities within programs implemented by India's large NGO sector.

This paper summarises findings from a participatory study that aimed to develop practical guidelines to make HIV programs in the states of Manipur and Nagaland more disability-friendly. Supported by the Research and Learning Fund of the UK Department for International Development (DFID), the one-year project (2006 – 07) was a collaboration between the University of Melbourne (Australia), the Emmanuel Hospital Association (EHA) (India), and research partners in both states. The objectives were to: 1) explore HIV risk and risk perception in relation to PWD among HIV and disability programmers, and PWD themselves; 2) identify HIV-related education and service needs and preferences of PWD; and 3) utilise findings and stakeholder consultation to draft practical guidelines for inclusion of disability into HIV programming in this region of India.

## Methods

### Study design

The study was undertaken in three phases and utilised various (mainly qualitative) tools and participatory approaches in each state. Participation of PWD themselves in every phase was a key feature of our methodology. PWD with a range of disabilities and socio-demographic backgrounds were sought to elicit data that reflected at least some of the diversity of this population in the two states. Because partnerships between disability and HIV/AIDS sectors were deemed essential to respond to PWD needs, individuals from these sectors also participated in these phases.

Too often, services and information campaigns are developed according to the assumptions of health programmers alone. Participatory formative research, using a range of methods, is essential to provide understanding of target needs as well as opportunities [23,24]. 'Participatory' research, according to some scholars, does not denote specific methods but rather an approach which emphasizes knowledge-for-action through local identification of problems, perspectives and priorities. This framework has been shown to produce more effective, sustained interventions, saving time and money [25]. Even epidemiologists have argued the merits of participation to overcome the limitations of conventional approaches:

*Creating community partnerships such that community representatives participate in the definition of the research problem, interpretation of the data, and application of the findings may help address these concerns*. [26]

Phase I involved a consultative workshop with PWD and providers of disability services to expand concepts and categories of enquiry, followed by Focus Group Discussions (FGDs) and semi-structured in-depth (individual) interviews (IDIs) with PWD and representatives of HIV and disability organisations. A structured survey (described below) was completed by HIV programmers. FGDs and IDIs were audio-taped and transcribed; transcripts were then translated into English and back-translated. Thematic analysis, based on the study objectives and the categories of enquiry, was used to analyse qualitative data, and simple descriptive statistics used to tabulate survey results.

Phase II consisted of a workshop in each state, attended by PWD and providers of both disability and HIV programs, to disseminate Phase I findings and construct a preliminary draft of the disability-friendly guidelines. These drafts were then refined with input from the international literature.

Phase III again involved convening workshops with the same range of participants (many were the same individuals) to elicit feedback from stakeholders on the draft guidelines. The guidelines have since been finalised and published.

The Australian-based investigators (MM and HD) trained local teams in unfamiliar concepts and methods, and attended and helped run the workshops. The Indian researchers conducted all FGDs and IDIs, and both sides shared the process of analysis and interpretation.

The study design was approved by the University of Melbourne's Human Research Ethics Committee and the EHA Institutional Review Board. All research participants were given information sheets about the study in which the confidentiality and voluntariness of participation was made clear. Informed consent was obtained from each participant (verbal for PWD and written for organisation representatives) and no inducements were given. A card with details about organisational affiliation and sources of further health information was also provided.

### Data collection tools

#### Survey questionnaire

An English-language structured self-report survey, comprising mainly closed (and several open) questions, was sent to representatives of HIV organisations in each state. The survey was adapted, with permission, from the *Yale/World Bank Global Survey on HIV/AIDS and Disability *[27]. It canvassed focal areas of work, perceptions of PWD vulnerability to HIV, and extent to which programs currently reach PWD.

#### Qualitative question guides

For FGDs and IDIs, semi-structured question guides, with main topics and secondary issues, were developed using the study objectives, a review of literature, and ideas generated during Consultative Workshops. These were translated into Nagamese and Manipuri, and then back-translated to ensure consistency.

#### Sampling, recruitment and process of data collection

The sample was exclusively urban, with participants recruited in the largest city in each state (Imphal, Manipur and Dimapur, Nagaland). PWD for FGDs and IDIs consisted of vision- and mobility-impaired individuals of both sexes (in equal numbers), aged 18 – 35. They were recruited purposively using these selection criteria, and identified through disability organisations, the Consultative Workshops, and snowballing. There were 2 – 3 refusals in each state. FGDs were organised separately by type of disability, sex and age (18 – 25 and 26 – 35). Participants for IDIs were purposively selected to expand the diversity of informants in relation to demographic background. Facilitators and interviewers were the same sex as the informant/s and used the local language.

HIV program providers were recruited for one FGD per state through the networks of Project ORCHID, an HIV-prevention program administered jointly by EHA and the University of Melbourne. Providers of disability programs (some of which addressed other types of disability besides vision- and mobility-impairment) were recruited for IDIs through the Consultative Workshops. FGDs and IDIs with providers were conducted in a mixture of English and the local language.

## Results

In each state four FGDs involving PWD and one FGD involving HIV providers were conducted. Five-six IDIs with PWD and three IDIs with representatives of disability organisations were also conducted. See Tables 1 and 2 for demographic breakdown of PWD sample for FGDs and IDIs, respectively. *(****Note****: the number of each FGD/IDI is used below to indicate the source of quotes*.)

**Table 1:** FGD demographics (PWD sample)

FGD no.	State	No. (sex) of participants	Type of disability	Age in years	Marital status	Educational level
1	Manipur	6 (F)	Blind	18–25	2 Married 1 Divorced 3 Unmarried	2 Undergraduate 1 Graduate 3 Secondary
2	Manipur	6 (F)	Physical	26–35	1 Married 4 Unmarried 1 Widow	4 Primary 2 Secondary
3	Manipur	7 (M)	Blind	26–35	2 Married 3 Unmarried 2 missing	4 Secondary 3 Undergraduate
4	Manipur	6 (M)	Physical	18–25	1 Married 5 Unmarried	4 Secondary 2 Undergraduate
6	Nagaland	5 (F)	Blind	18–25	5 Unmarried	Missing
7	Nagaland	6 (F)	Physical	26–32	1 Married 5 Unmarried	All literate
8	Nagaland	6 (M)	Blind	26–35	3 Married 3 Unmarried	2 Illiterate 2 Secondary 1 Higher secondary 1 Undergraduate
9	Nagaland	6 (M)	Physical	18–25	6 Unmarried	2 Primary 1 Secondary 2 Higher secondary 1 Undergraduate

**Table 2:** IDI demographics (PWD sample)

IDI no.	State	Sex	Type of disability	Age in years	Marital status	Educational Level
M 1	Manipur	F	Blind	26	Unmarried	Secondary
M 2	Manipur	F	Physical	35	Unmarried	Higher secondary
M 3	Manipur	F	Physical	35	Widow	Higher secondary
M 4	Manipur	M	Blind	28	Married	Secondary
M 5	Manipur	M	Physical	18	Unmarried	Secondary
M 6	Manipur	M	Physical	34	Unmarried	Secondary
N 1	Nagaland	F	Physical	27	Unmarried	Higher secondary
N 2	Nagaland	F	Physical	23	Unmarried	Illiterate
N 3	Nagaland	M	Blind	35	Unmarried	Higher secondary
N 4	Nagaland	M	Physical	28	Unmarried	Undergraduate
N 5	Nagaland	M	Physical	21	Married	Illiterate/No school

A total of 52 surveys (25 from Manipur, 27 from Nagaland) were completed and returned by representatives from 32 HIV/AIDS organisations (16 per state). These representatives were coordinators of programs.

Analysis revealed a number of challenges, needs, preferences and opportunities for responding to HIV-related risk and vulnerability of PWD living in Nagaland and Manipur.

### Challenges in finding and reaching PWD and responding to the needs of PWD

There are practical and sociocultural difficulties in identifying, locating and reaching PWD that arise directly from the context and life circumstances of this population in northeast India. Many PWD are housebound and thus less likely to access mainstream programs or services. This arises for a variety of reasons: terrain, weather, lack of mobility aids; being single and female in a traditional setting; and fearfulness prompted by experiences of stigma or harassment. Lack of comprehensive population data that includes PWD adds to their 'invisibility' to outreach service providers.

A sense of the social isolation and exclusion experienced by PWD was evident in many FGDs and IDIs. Gender discrimination plays a role, as do fear of the different, and ancient superstitions, e.g., in Manipur, where pregnant women avoid gazing upon the disabled for fear their foetus will be deformed.

*Now she has been gradually losing her eyesight just like me. So, her mother hides her. Thinking that the girl could never get married because of her disease, her mother did not allow her to have a proper education*. (FGD 1) *(****Note***: *The number indicates the source of the quote and corresponds to *Tables 1 and 2.)

*Sometimes I feel, "What is the use of going out with others?" Sometimes I feel ashamed. I am not like others...so I don't go out*. (IDI N 2)

*Those days, when I was doing something, when I walk, they said "why do you have to [appear in public]?" *(IDI M 2)

One of the most striking challenges for provision of HIV programs to PWD is that most respondents routinely rely on carers, hostel wardens, and family to facilitate their participation in the wider community. These 'gatekeepers' ordinarily are protective and well-intentioned, but may not recognise the potential HIV vulnerability or information needs of PWD, or may be hostile to the very notion as a slur on the morals of individuals under their care.

*Even when one knows that nothing will happen they will not bring out their child thinking that others will tease or make fun of them*. (IDI Manipur disability service provider with intellectually impaired)

A final challenge is the risk inherent in generalising about 'PWD', who in fact vary greatly according to key sociodemographic factors, e.g., education, marital status, sex, geographic location and occupation, as well as the nature and severity of disability, and whether congenital or acquired. This heterogeneity has implications for meeting the needs of individuals.

*It depends even on economy of family and relations [of PWD]. If well-to-do, [they have] more chance of getting services*. (FGD 9)

*For the blind, they can easily hear and learn ... The deaf are the ones who face the biggest problem. For [them ... whatever] is said, informed, will not be heard*. (FGD 4)

### HIV knowledge and awareness among PWD

FGDs and IDIs revealed much diversity in levels of HIV understanding and awareness among the sample. Those with visual impairments had more education and greater HIV knowledge than their physically impaired counterparts. Overall, males were also more knowledgeable than females. Two participants had *'never heard of HIV/AIDS'*. Notably, some representatives from disability organisations also demonstrated incomplete knowledge and a lack of awareness of HIV and appropriate prevention strategies.

When prompted to identify HIV transmission risks, most informants mentioned needle sharing. Unprotected sex was less commonly cited, and no one mentioned male-male sex. Some of their more worrying misconceptions hark back to myths popular in the epidemic's early days, e.g., 'mosquitoes transmit HIV', and the 'greater' risk of infection from '*having sexual contact with unknown persons' *(IDI M4). Some saw risk in sharing of utensils and clothing, or contact with menstrual blood, while one spoke of the role of divine intervention: *'We should go to the church and the healing power can [cure HIV]' *(FGD 6) There was some evidence that HIV education about transmission via blood has been conflated with traditional Manipuri concepts and terminology, where 'blood group' indicates degree of relationship. One young blind Manipur woman suggested, '*But if [their blood groups] are not compatible, the other will not get infected*.' (IDI M 1)

### PWD risk and vulnerability: perceptions and experiences

Most study participants of all types, including 45 of the 52 HIV programmers who completed the survey, agreed that PWD living in Nagaland and Manipur may be vulnerable to HIV. Although PWD participants were more likely to cite needle-sharing than sex as an HIV risk behaviour in general populations, when asked to consider potential vulnerability for PWD most felt sexual transmission was the likeliest mode. However, most spoke about this obliquely and with evident embarrassment. A few also mentioned possible transmission among PWD who use injection drugs, but no one cited the risk of family sexual abuse as a potential transmission route for PWD.

Most also felt that the degree of vulnerability was associated with the type and severity of disability, along with sociodemographic factors. However, these views often emerged slowly over the course of an FGD/IDI, after the discussion or clarification of HIV transmission routes, and in response to prompt questions about potential risks. During the Nagaland Consultative Workshop, one disability provider strenuously objected to the notion that PWD could be vulnerable, but others rebutted his arguments. In the Manipur Workshop, several disability providers expressed shame at not having recognised the risks for their clients, which now – due to the workshop – struck them as obvious.

There was ready recognition of the potential transmission risk for intellectually disabled people of both sexes, whose lack of inhibitions, awareness and capacity to communicate make them *'soft targets.'*

*A women who is old, age-wise, but remains childish in her behaviour because of her mental abnormality ... people take advantage of her mental disability. So, this is a risk factor*. (FGD 1)

Many believed that hearing-impaired women are often victims of sexual assault, and said some enter the commercial sex industry.

*Mute and deaf are more vulnerable among the disability types. There is a story that a man likes women who are mute and deaf. Here, the man ultimately tries to have sexual contact...he even wants to rape the women*. (IDI M4)

Both vision- and mobility-impaired informants, particularly males, felt those whose disability did not greatly constrain their mobility were likeliest to engage in high risk behaviours, and some drew on their own experiences.

Many homeless people in these states are disabled, and this group was identified as vulnerable on two fronts.

*Some others are just roaming in the streets, sleeping in the streets, street women, beggars – this group have more chance of getting infected with HIV/AIDS through raping by some other beggars, rag-pickers*... (FGD Nagaland HIV organisations)

Disability service providers also mentioned the risk of unprotected male-male sex among those living in hostels for the blind or in institutions and lacking access to information.

*We can consider those people who stay away from home and who stay among the same sex for long as a risk group... They are at risk because they are at the experimenting phase and then biologically they are sexually active... Unluckily they are not given proper sexual education. There is also no policy*. (IDI Manipur disability service provider with intellectually impaired)

The double discrimination of gender and disability renders female PWD more vulnerable than their male or non-disabled counterparts, according to some informants. Several blind participants expressed high levels of anxiety about leaving home on this account, with one telling of a blind woman who *'went insane' *after being raped by an auto-rickshaw driver.

*Unmarried blind females should be more cautious..... We may either be trapped or kidnapped by other people because we do not see anything*. (FGD 1)

*As for us women... we say 'yes' most of the time*. (IDI M3)

Some participants also suggested that PWD in these states may be more likely to engage in transactional sex due to unemployment and economic disadvantage.

Participants were not asked directly about their own risk behaviours. A small number did volunteer that they had shared needles when injecting drugs or engaged in unprotected sex with sex workers, and two stated that they were HIV-positive. Survey results demonstrated that there are other HIV-positive PWD known to organisations in these states. Participants spoke feelingly about the enormous stigma likely to be experienced by those carrying a double burden.

*He's disabled and on top of that he is infected with a dreaded disease. I think people will look down upon him with contempt and disgust*. (IDI M1)

The traditionally low status of widows, especially in Hindu communities, is exacerbated for those whose husbands died of drug abuse or HIV/AIDS; this is amplified for PWDs. A widow in our sample, infected by her IDU husband, put it this way:

*The wives get infected from their husbands but the wives are blamed... [my in-laws] didn't like me before [he died] and if they know that I am infected, they'll despise me more*. (IDI M3)

### PWD programming needs and preferences

At present, HIV programmers in these states only reach PWD by chance when they happen to fall within their catchment area, and there are no HIV prevention or care programs run by disability service providers. We asked PWD to identify their perceived needs, as well as the avenues and approaches they prefer for receiving such programs.

Many PWD participants felt that mainstream HIV messages and services were unlikely to reach the majority of PWD living in Nagaland and Manipur. Various reasons were cited, e.g., being 'hidden' and housebound, the role of gatekeeper, non-engagement in the community, perceived inconvenience or stigma related to seeking services, and type of disability. It was suggested that an outreach service may be an appropriate way to identify PWD in the community and educate and develop trust with gatekeepers, who play a crucial role in providing access, as well as protection for the most vulnerable PWD.

*Because disabled, unlike other people, are not so active, if someone can come and give information and services it will be good*. (IDI N4)

*I think [providing service to intellectually-impaired] is going to be a bit difficult. Since they cannot understand...the parents have to be contacted first. We have to talk to the parents*. (IDI M3)

Participants highlighted the crucial aspects of disability type and levels of literacy in relation to program preferences. For the minority of people with visual impairments who are literate, HIV educational materials in Braille format were thought useful, whereas the non-literate need radio and TV messages. Most people with hearing impairments in these states cannot read or use sign language; service providers felt simple visual materials and education of gatekeepers were most desirable. The high rate of illiteracy among PWD in general creates a need for varied and creative approaches.

*Posters can be effective. But ... for the uneducated and the illiterates posters would be meaningless. There should be meetings, seminars and conventions in this regard*. (IDI N4)

Most PWD expressed a preference for peer education, but acknowledged that the peer educator must be well informed and supported through appropriate organisations.

*A disabled telling a disabled would be good...in this case communication would be effective*. (IDI N5)

*That kind of activity needs skill on the part of the person who has to handle the disability ... I think that would be very good because he himself would be very happy when he is given such a role. To the disabled community he would become a role model*. (IDI M6)

Participants cited the need for HIV services to be physically accessible, e.g., on the ground floor, with chairs available, and ramps for wheel chairs. Some mentioned the importance of having assistants to provide greater confidentiality for individuals who would otherwise rely on family or friends to access such services. Several women expressed a preference for separate and specialised HIV services, but others feared this could increase the stigma faced by PWD by implying that PWD are a group that engages in HIV risk behaviours.

While PWD acknowledged the existence of disability service providers, many expressed cynicism about their willingness and capacity to be useful, citing examples of indifference and even suspected corruption, particularly in the government sector. Such claims cannot be verified, but these perceptions are significant for decision-making about the most appropriate ways to meet PWD needs and preferences for HIV programs.

### Challenges faced by HIV programmers in meeting PWD needs and preferences

While the HIV programmers who participated in FGDs and workshops expressed great empathy for the needs of PWD, they emphasised the difficulties in responding. For one thing, many organisations have been established with specific mandates to serve recognised risk groups, particularly IDUs and sex workers. For another, they are often struggling to meet their work requirements – typically with very low budgets – and most felt it beyond their capacity (human and financial) to address a new, complex sub-group. HIV programmers in Nagaland described their challenges as follows:

*We are directed in such a way that [the] targeted group is to be looked after and we have to do according to the direction, so we don't have such resources for all such [extra] things... Because the services we provide now is for sex workers, IDUs, [men who have sex with men], transgender also, these are the categories we are looking after, but disabled-handicapped category is not there... I think we must be trained. But training is also not enough. We have to meet their needs and to do that we need financial assistance*. (FGD Nagaland HIV organisations)

## Discussion

This research among PWD and providers of disability and HIV programs in Manipur and Nagaland found that participants perceive PWD to be vulnerable to HIV infection, but the level of vulnerability or risk depends upon type and severity of impairment, gender and socioeconomic circumstances. PWD reported widespread experiences of humiliation and stigma connected to disability, but exacerbated by the demographic factors that independently confer or reduce social status, such as education, occupation, living situation and gender norms. There was evidence showing connections between stigma and HIV vulnerability, as well as access to HIV prevention and care.

Many PWD participants have misconceptions regarding prevention and management of HIV infection. Similar findings have been reported from studies involving PWD in the African continent [11,12,28,29]. Not surprisingly, male participants (who are more mobile and engaged in society than females) and those with higher educational levels had more accurate knowledge. It is well-known that formal education can have a protective effect in HIV transmission among non-disabled populations [1,30-32]. Mulindwa [8] also demonstrated that condom use among people with visual, hearing and movement impairments living in Uganda increased with level of educational achievement. Clearly, general education and education about HIV are needed among PWD everywhere, including in northeast India.

Cultural taboos limit the free discussion of sexual issues in Indian society [21,33,34]. The embarrassment shown in discussing this topic even within same-sex group discussions, and the absence of reference by PWD to male-male sex, points to the need for explicit messages about risk practices. As most HIV prevention materials use indirect language and do not touch upon the most sensitive topics, such as male-male sex and sexual abuse, there are worrying gaps for PWD. Given the reality of sexual abuse of PWD (especially the hearing- and intellectually-impaired), it is essential that the wider community recognise its role in protection and prevention.

PWD in these states face social exclusion and poverty, which in turn reduce their access to mainstream HIV information and services. Research elsewhere in India has demonstrated that PWD experience difficulty accessing a variety of community services, including health services, due to: lack of awareness of their rights and available programs [35]; physical barriers limiting effective access; and the attitudes of health professionals and the wider community [36]. A recent national survey revealed substantial disadvantage among PWD, finding 55% were illiterate, only 9% had achieved secondary schooling, and just 26% were employed [37].

Another crucial factor is the role of the 'gatekeeper', whose own level of knowledge of HIV, and awareness of the vulnerability of those under their care, can determine what information reaches these individuals. The understandable tendency for guardians and family members to be protective may paradoxically reinforce actual and perceived exclusion among PWD. Guardians may also fail to recognise the information and service needs of PWD, who are often (sometimes incorrectly) perceived as not sexually active [9-11,13,27]. Cultural taboos in India may also inhibit transfer of information. Because many HIV programmers deal directly with target populations, the idea of an indirect approach (to or through gatekeepers) may present challenges for design of interventions.

The expressed preference for peer outreach undoubtedly reflects anxiety about humiliation and discrimination felt by many PWD. Peer outreach has been utilised by HIV organisations in other parts of India to address the needs of minority groups in rural areas and sex workers [38,39]. Training and involvement of PWD as peer educators would also increase their skills and self-esteem, and help break down barriers between PWD and the general population. Other mechanisms that help PWD feel comfortable and safe in seeking services are crucial to their uptake; modification of premises, chairs for waiting, and designated assistants who make the involvement of a family member unnecessary (and thus help ensure confidentiality), may be instrumental. Development and dissemination of information in a variety of formats and messages are also needed to enable individuals with different types of disability to achieve their basic right to health information.

No HIV prevention or care interventions especially for PWD exist at present in these states. There are practical, financial and logistical obstacles for HIV programmers and disability service providers alike, but there is emerging enthusiasm – expressed during workshops within this project – for meeting the needs of PWD within this high prevalence setting.

### Study limitations

This research had a number of limitations. Firstly, the design and budget did not permit the recruitment of a random sample of PWD. While this was addressed partially through consultations with disability organisations, the findings cannot be generalised to the population of PWD in these states. Moreover, participants were recruited through disability organisations, which resulted in some selection bias towards the more educated. It should be noted, however, that our findings are broadly similar to those from studies conducted with PWD in other settings. Secondly, due to the technical, logistical and ethical challenges of including individuals with hearing and intellectual impairments, as well as mental illnesses, the findings relate primarily to those with physical or visual impairments. Research has identified that people with mental illness in India may engage in high risk behaviours and be at risk of sexual coercion [40,41]. Thirdly, the method used to approach HIV organisations for completion of the survey resulted in multiple submissions from some but not from others, thus ruling out comparability between states or organisations. Finally, this study did not seek to produce quantitative data on HIV prevalence or risk behaviours among PWD, although some evidence about these emerged. The acute lack of data in this area underscores a need for further research.

### Development of guidelines

On the basis of study findings, workshops and stakeholder feedback, the authors have published and distributed in India a document entitled: *Guidelines to Foster Disability-Friendly HIV/AIDS Programs: For use by the HIV/AIDS and Disability Sectors in Manipur and Nagaland *[42]. The majority of recommendations utilise existing structures and services and involve adaptations which would not require significant additional economic resources. Key recommendations include:

• Creating networks between HIV and disability organisations, so that their respective specialist skills can be shared to enable a rapid, and effective, response to the needs of PWD;

• Providing materials in a variety of formats and disseminating these through the disability sector and pre-existing social institutions;

• Targeting gatekeepers for awareness-raising;

• Engaging and educating community and religious leaders on the HIV vulnerability and needs of PWD;

• Providing transportation, allowances and outreach to improve access for PWD to mainstream services;

• Promoting the right to education for PWD to reduce their vulnerability due to socioeconomic circumstances; and

• Considering specialist HIV services for people with specific types of disability who cannot be reached with adaptations to mainstream services.

Specific suggestions for responding to the needs of people with visual, hearing, physical and intellectual impairments were also made in these guidelines.

## Conclusion

The research component of this project found that some PWD living in Nagaland and Manipur may be vulnerable to HIV infection due to a lack of awareness and knowledge of HIV transmission, social exclusion and poverty. This project has provided HIV and disability organisations with practical guidelines and new networks, which increase the potential to meet the HIV education and service needs of PWD in these states. The increasing HIV prevalence among the general populations in Nagaland and Manipur calls for an 'integrated and all-inclusive' response [43]. Greater visibility and activism of disability organisations is important for advocacy. Under NACP III and through the State AIDS Committees there is an important opportunity to address the needs of a neglected, and very disadvantaged, sub-group of the Indian population. This is essential from both a public health and human rights perspective.

## Abbreviations

EHA Emmanuel Hospital Association

DFID (UK) Department for International Development

FGD Focus group discussion

IDI In-depth interview

IDU Injecting drug user

NACP National AIDS Control Programme

NGO Non-governmental organisation

PWD People with Disability

## Competing interests

The author(s) declare that they have no competing interests.

## Authors' contributions

MM and HD designed the study and trained the Indian-based research team. MM took the lead on designing of qualitative research tools, analysing qualitative data, interpreting data sets, and revising and finalising the manuscript. MCA led the Indian-based research teams and assisted with data analysis and interpretation. EP conducted a literature review for this paper and wrote the initial draft based on summary analysis documents prepared by the other authors. All authors read and approved the final manuscript.

## Pre-publication history

The pre-publication history for this paper can be accessed here:


